# Optimizing the Removal of Rhodamine B in Aqueous Solutions by Reduced Graphene Oxide-Supported Nanoscale Zerovalent Iron (nZVI/rGO) Using an Artificial Neural Network-Genetic Algorithm (ANN-GA)

**DOI:** 10.3390/nano7060134

**Published:** 2017-06-03

**Authors:** Xuedan Shi, Wenqian Ruan, Jiwei Hu, Mingyi Fan, Rensheng Cao, Xionghui Wei

**Affiliations:** 1 Guizhou Provincial Key Laboratory for Information Systems of Mountainous Areas and Protection of Ecological Environment, Guizhou Normal University, Guiyang 550001, China; xuedanshi1991@163.com (X.S.); wenqianruan@yahoo.com (W.R.); fanmingyifmy@163.com (M.F.); 18230825324@163.com (R.C.); 2 Department of Applied Chemistry, College of Chemistry and Molecular Engineering, Peking University, Beijing 100871, China; xhwei@pku.edu.cn

**Keywords:** rhodamine B, nanoscale zero-valent iron, graphene, RSM, ANN-GA, adsorption isotherm and kinetics

## Abstract

Rhodamine B (Rh B) is a toxic dye that is harmful to the environment, humans, and animals, and thus the discharge of Rh B wastewater has become a critical concern. In the present study, reduced graphene oxide-supported nanoscale zero-valent iron (nZVI/rGO) was used to treat Rh B aqueous solutions. The nZVI/rGO composites were synthesized with the chemical deposition method and were characterized using scanning electron microscopy (SEM), X-ray diffraction (XRD), Raman spectroscopy, N_2_-sorption, and X-ray photoelectron spectroscopy (XPS) analysis. The effects of several important parameters (initial pH, initial concentration, temperature, and contact time) on the removal of Rh B by nZVI/rGO were optimized by response surface methodology (RSM) and artificial neural network hybridized with genetic algorithm (ANN-GA). The results suggest that the ANN-GA model was more accurate than the RSM model. The predicted optimum value of Rh B removal efficiency (90.0%) was determined using the ANN-GA model, which was compatible with the experimental value (86.4%). Moreover, the Langmuir, Freundlich, and Temkin isotherm equations were applied to fit the adsorption equilibrium data, and the Freundlich isotherm was the most suitable model for describing the process for sorption of Rh B onto the nZVI/rGO composites. The maximum adsorption capacity based on the Langmuir isotherm was 87.72 mg/g. The removal process of Rh B could be completed within 20 min, which was well described by the pseudo-second order kinetic model.

## 1. Introduction

Dyes and pigments constitute a very important class of water pollutants due to their large scale usage in various industries, such as textile, printing, plastics, and leather, which can lead to water coloration [1,2]. Since small quantities of dyes and pigments in effluents may be toxic and highly visible, great attention has been given to the effluents containing these hazardous chemicals following the increase in the discharge pressure on the environment directly or indirectly [3,4]. Rhodamine B (Rh B) is a synthetic basic dye imparting red color in aqueous solutions that is widely used as a colorant in the textile and food industries, and also for cell fluorescence staining in the laboratory [2,5,6]. It has been reported that Rh B is harmful to the environment, humans, and animals, because of its carcinogenicity, reproductive and developmental toxicity, neurotoxicity, and chronic toxicity [7,8,9,10,11,12]. A wide range of methods can remove dyes from effluents, such as physical adsorption, chemical degradation, biological degradation, photodegradation, or the synergic treatments of different methods [13]. 

Currently, a growing number of studies have reported the application of nanoscale zero-valent iron (nZVI) for environmental remediation due to its high effectiveness, versatility, and unique properties, such as large specific surface area, small particle size, excellent reactivity, and high injectability into an aqueous solution [14,15,16]. It is essential to maintain the size, crystal form, and morphology of nanomaterials by controlling their preparation methods, such as temperature, solvent, and stabilizing agent [17]. Bare iron nanoparticles tend to rapidly agglomerate and oxidize and then a core-shell structure is formed. This can partially decrease the reduction of nZVI, but may work as an efficient adsorbent [18]. Previous studies have reported that nZVI was supported on materials to maintain the stability and mobility of nanomaterials, e.g., resin [19], chitonsan [20], activated carbon [21], and Kaolinite [22]. In addition, graphene is an ideal carrier for loading nanoparticles, which has a unique two-dimensional planar structure and a high specific surface area. A range of earlier studies have discussed the reduced graphene oxide-supported nanoscale zero-valent iron (nZVI/rGO) composites, which have been considered simple and effective for the treatment of contaminants, such as cadmium, uranium, arsenic, and trichloroethylene [23,24,25,26,27]. To our knowledge, hitherto there are no reports available in the literature regarding Rh B removal by nZVI/rGO composites.

The classical way to predict the adsorption process originates from a deep understanding of adsorption mechanisms. The unavoidable simplifications and assumptions for some highly complex processes can lead to inaccurate predictions during the development of mechanistic models [28,29]. Both the empirical and statistical models can be utilized for the known data to extract knowledge about operating conditions without knowing the sophisticated underlying mechanism of the process [28]. The conventional approach for optimization of process variables obviously requires a very large number of experiments to be performed, which would be very expensive and time consuming [2]. It would not be able to reveal the influence of the interactions between the process variables on the dependent variable [30]. To achieve optimal control and management of Rh B pollution, new concepts involving efficient operation and design should be developed and understood [31]. The experimental design technique is a statistical method that uses quantitative data from appropriate experiments to determine regression models and operating conditions [32]. Hence, response surface methodology (RSM), including Box-Behnken design (BBD) and central composite design (CCD), has been widely applied for the optimization of chemical and physical processes [33,34], and the number of experiments can be decided accordingly. In addition, a host of data analysis tools have been developed into well-established modeling techniques, such as: fuzzy logic (FL) and artificial intelligence (AI). Artificial neural networks (ANNs), as one of the major tools for AI, have been widely accepted as a technology to which is attributed its universal ability to simulate non-linear variations and handle incomplete data [35,36]. ANN can be an effective tool for predicting and optimizing any complex process parameters, but does not guarantee the global optimal solution [36]. Hybridizing ANN with genetic algorithms (GA) is an effective method for process modeling and optimization, which can generate optimum operating variables for the studied process [37,38].

The major objective of the present study is to investigate the feasibility of using nZVI/rGO for the removal of Rh B from aqueous solutions. At first, the nZVI/rGO composites were prepared by using the chemical deposition method, and different analytical techniques were used to characterize the nZVI and nZVI/rGO materials. Then, the process parameters (initial pH, initial concentration, temperature, and contact time) for the optimum removal capacity of Rh B were obtained with the RSM and ANN-GA models. For the ANN model, the number of neurons was selected based on the mean square error (MSE). Finally, batch experiments were carried out to study the adsorption isotherm models and kinetics models.

## 2. Materials and Methods

### 2.1. Materials

All reagents and chemicals used (NaBH_4_, H_2_SO_4_, HCl, NaOH, and FeSO_4_·7H_2_O) in this study were of AR grade. Rhodamine B (Rh B) was obtained from Tianjin Kemio Chemical Co., Tianjin, China. Stock solution of Rh B (1000 mg·L^−1^) was prepared by dissolving in deionized water, which was further diluted with deionized water to the required initial concentrations (60, 80, 100 mg·L^−1^). Graphite powder (<30 μm) was supplied by Sinopharm Chemical Reagent (Beijing, China). Absorbance measurements were made on a UV-Visible spectrophotometer (Cary 100 Bio, Varian, PaloAlto, CA, USA).

### 2.2. Preparation of nZVI and nZVI/rGO

Graphene oxide (GO) was synthesized from graphite using the modified Hummer’s method [39,40,41,42]. In the typical reaction for the synthesis of nZVI-rGO, 1.0 g of GO was added into 300 mL deionized water by a 2 h ultrasonication bath, and 50 mL of an aqueous solution of 10.0 g FeSO_4_·7H_2_O in water was injected into the GO suspension stirred slowly by a magnetic stirrer for 12 h. After that, NaBH_4_ solution (5.46 g/50 mL) was added dropwise to the mixture and stirred for 30 min. The black precipitate was collected by vacuum filtration and washed several times with de-ionized water and ethanol, and finally dried at 50 °C in vacuum. The nZVI was prepared similarly to nZVI/rGO, without the addition of graphene oxide.

### 2.3. Characterization of nZVI and nZVI/rGO

Scanning electron microscopy (SEM, Quanta F250, FEI, Hillsboro, OR, USA) were performed to study the surface characteristics and morphology of the nZVI and nZVI/rGO. X-ray diffraction (XRD) was carried out on a Philips Analytical X-ray (Lelyweg 1 7602, EA, Almelo, The Netherlands) with a Cu-Kα X-ray source (generator tension 40 kV, current 40 mA). Raman spectra of nZVI and nZVI/rGO were recorded at 532 nm by Raman spectroscopy (LabRAM HR800, Horiba Jobin Yvon, Paris, France). Specific surface areas of nZVI and nZVI/rGO were obtained using the Brunauer-Emmett-Teller (BET, Quadrasorb SI, Quantachrome Instruments, Boynton Beach, FL, USA) methods from the corresponding N_2_ adsorption/desorption isotherms at 77 K. The nZVI and nZVI/rGO composites were characterized by X-ray photoelectron spectroscopy (XPS) and the peak energies were corrected with the C1s peak at 284.8 eV as a reference.

### 2.4. Batch Experiments

For each experimental run, 0.04 g of nZVI/rGO was added into 50 mL of Rh B solution with a known initial pH and the initial concentration (C_0_), which was taken in a 250 mL stoppered conical flask. The mixture was shaken on a temperature controlled water bath shaker at 200 rpm for the desired temperature and contact time. The initial pH value of the sample solution was adjusted by the addition of 0.1 mol/L HCl or 0.1 mol/L NaOH to conduct the batch experiments at the desired pH.

The nZVI/rGO was separated from solution for all of the samples, and the Rh B concentration in solution was analyzed by a UV-Visible spectrophotometer at *λ*_max_ of 552 nm. A blank experiment with only the adsorbent in 50 mL of deionized water was conducted simultaneously at similar conditions to account for any color leached by the adsorbent and adsorbed by the glass containers. The removal percentage and the removal capacity of Rh B were calculated using Equations (1) and (2).

(1) $$Y=(C_{0}-C_{t})/C_{0}\times 100$$

where *Y* is the removal percentage of Rh B, *C*_0_ is the initial concentration of Rh B ions (mg/L), and *C*_t_ is the concentration of Rh B after removal.

(2) $$q_{e}=(C_{0}-C_{t})\times V/m_{s}$$

where *q_e_* is the removal capacity of Rh B (mg/g), *V* is the volume of the solution used, and *m_s_* is the weight of nZVI/rGO (g).

### 2.5. Box-Behnken Design

As is well known, orthogonal design can only analyze the experiment points, while BBD can continuously analyze the various levels of the experiment, which is a class of three-level (coded as −1, 0, and +1) factorial designs. Clearly, BBD is a time and money-saving method due to less experiments being required [43]. In addition, this method is able to show the interaction effects of variables, which help to better understand and improve the efficiency of the process [44]. In the present work, BBD was applied to evaluate the interactive effects for Rh B removal by nZVI/rGO. The initial pH (*A*), initial concentration (*B*), temperature (*C*), and contact time (*D)* were selected as independent variables for the determination of the optimal conditions, and the removal percentage of Rh B (*Y*) was considered as the dependent variable. The number of experiments needed for optimization of the removal process was reduced from 81 to 29 by using BBD. The data obtained from the experiments were analyzed using the coefficient of determination (R^2^), Pareto analysis of variance (ANOVA), and 3D response surface plots [45]. A non-linear regression method was used to fit the second degree response surface expressed as below:

*Y* = *X*_0_ + *X*_1_*A* + *X*_2_*B* + *X*_3_*C* + *X*_4_*D* + *X*_5_*AB* + *X*_6_*AC* + *X*_7_*AD* + *X*_8_*BC* + *X*_9_*BD* + *X*_10_*CD* + *X*_11_*AA* + *X*_12_*BB* + *X*_13_*CC* + *X*_14_*DD* (3)

where *Y* represents the removal percentage of Rh B, *X*_0_ is a constant offset term; *X*_1–14_ are the estimated coefficients, and *A, B*, *C* and *D* are coded factors (Table 1).

### 2.6. ANN Modeling and ANN-GA Optimization

**Figure 1:**
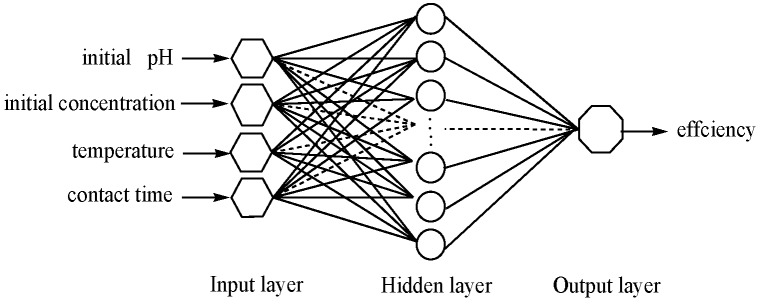
Simple illustration of the artificial neural network (ANN) structure.

**Figure 2:**
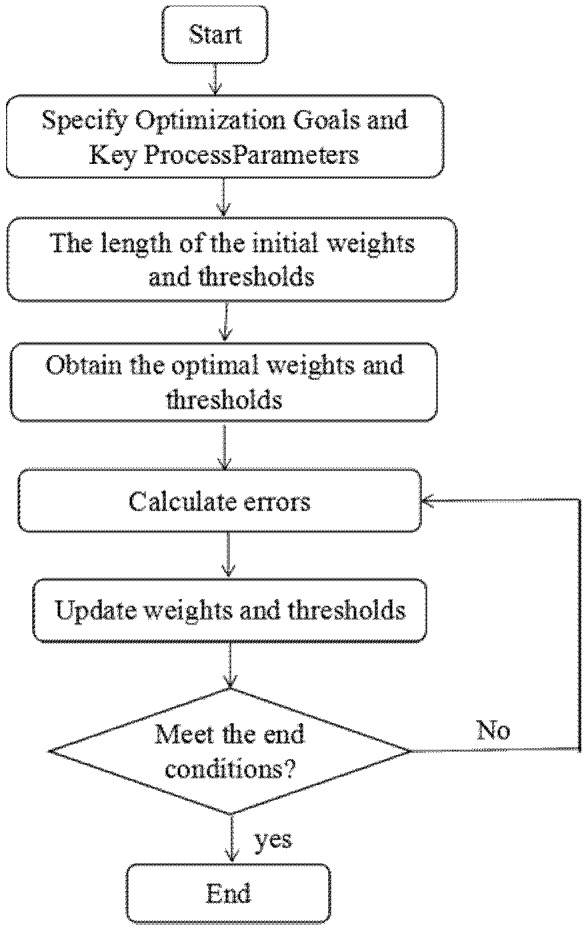
Flow diagram of ANN training.

ANNs are from direct inspiration from the biology of neural systems. ANN and ANN-GA combination were applied to build the predictive mathematical model and optimization, using the neural network and genetic algorithm toolboxes by MATLAB (Version 2010a) [35]. The ANN model was performed by using the back propagation (BP) algorithm, which is a gradient descent optimization procedure [35,38]. The RSM data (29 sets) used for the ANN network training was divided into training groups (1–24 sets, 80%) and a validation group (25–29 sets, 20%). The number of neurons in the hidden layer is in direct proportion with the fitting performance of the network. Therefore, the model responses were compared to the experimental data to calculate the MSE, which was used as a criterion in relation to the number of neurons in the hidden layer [46]. Tansigmoid and purelin were used as transfer functions in the hidden and output layers, respectively. The simple illustration of the ANN structure is shown in Figure 1. The established artificial neural network was trained with the traingdx algorithm, which improves the leaning speed and increases the reliability. The Traingdx method running for 2000 epochs with the learning rate at 10^−5^ and minimization is with respect to a defined MSE. The flow diagram of ANN training is shown in Figure 2.

All values of the inputted variables for the ANN model were normalized between −1 and +1 using the following equation:

*X_i_* = 2 × (*X* − *X*_min_)/(*X*_max_ − *X*_min_) − 1 (4)

where *X_i_* stands for normalized values, and *X*, *X*_min_, and *X*_max_ are the original, minimum, and maximum values of variables, respectively.

**Figure 3:**
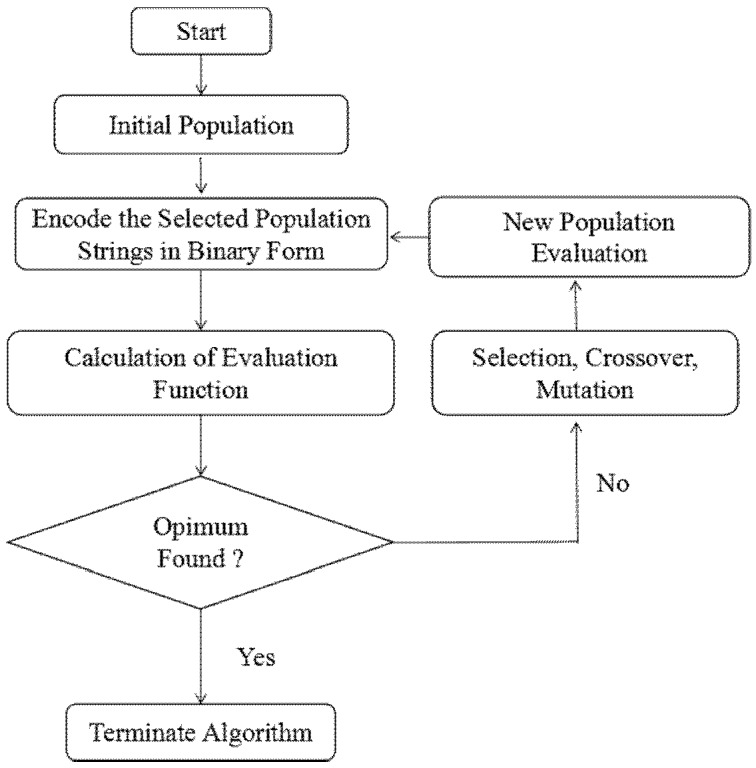
Flow diagram of genetic algorithm (GA).

Moreover, the input variables of the model become the decision variables for the optimal operating conditions and the highest Rh B removal efficiency using GA. The use of GA starts with the creation of the blind random population defined by the problem at hand [36]. In the genetic algorithm, variables are known as “chromosomes”, and each chromosome was made up of a distinct media composition consisting of four different genes (individual = pH, initial concentration, temperature, contact time = *x_1_, x_2_, x_3_, x_4_*). A group of chromosomes are called a population. The algorithm runs more slowly with a large population size, thereby a suitable population size numbers about 20–30 chromosomes [35,47,48]. The roulette wheel scheme was used to determine a string with a higher chance of surviving in subsequent generations [48]. The fitness function was applied to optimize the Rh B removal in the ranges of the process variables. The values of the GA parameters for population size, number of generations, crossover rate, and mutation probability were 20, 100, 0.8, and 0.01, respectively. A simple explanation of GA is shown in Figure 3.

## 3. Results and Discussion

### 3.1. Characterization of nZVI and nZVI/rGO

**Figure 4:**
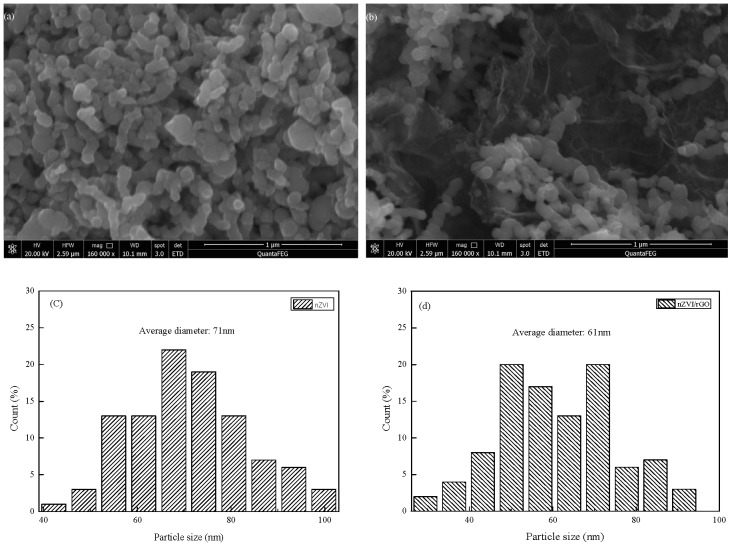
Scanning electron microscopy (SEM) images of nanoscale zero-valent iron (nZVI) (**a**) and nZVI/ graphene oxide (rGO) (**b**); Size distributions calculated from the SEM images for nZVI (**c**) and nZVI/rGO (**d**).

The SEM images of nZVI and nZVI/rGO are shown in Figure 4a,b. The surface morphology of the nZVI nanoparticles is aggregated together tightly, as shown in Figure 4a. From Figure 4b, it is seen that nZVI can be dispersed well on the reduced graphene oxide (rGO) sheet. It is dispersed more homogeneously and predominantly than the pure nZVI, however there is still some agglomeration. The diameter distribution of nZVI and nZVI/rGO is concentrated in the range of 40–105 and 20–95 nm, and the average diameter of nZVI and nZVI/rGO is 71 and 61 nm, respectively (Figure 4c,d). This also indicated that the rGO can be dispersed in the nZVI particles.

**Figure 5:**
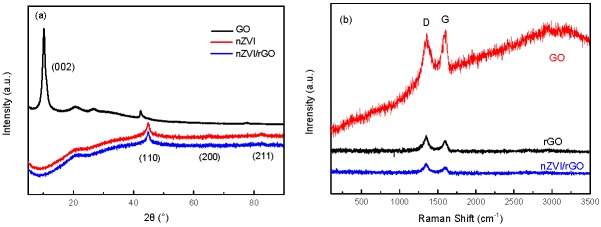
X-ray diffraction (XRD) patterns of nZVI and nZVI/rGO (**a**); Raman spectra of GO, nZVI, and nZVI/rGO (**b**).

The X-ray diffraction patterns of GO, nZVI, and nZVI/rGO are shown in Figure 5a. The characteristic peak at the 2θ value of 10.9° (002) confirmed that the graphite powder was well oxidized to graphene oxide. This peak disappeared in nZVI/rGO because of the changes to the packing of the rGO sheets by the presence of the nZVI particles. The XRD patterns for the nZVI and nZVI/rGO composite reveal the representative peak of the α-Fe^0^ at the 2θ angles of 44.7°, 65.0°, and 82.6°, indexed to the diffraction peaks from (110), (200), and (211), respectively [16]. These peaks were consistent with the standard XRD data for the body-centered cubic (BCC) Fe^0^ (JCPDS No. 010-87-0722).

According to the Raman spectra (Figure 5b), the D peaks (defects and disorders caused by the sp^3^ carbon atoms) and G peaks (sp^2^ carbon atoms in the graphitic sheets) are observed for GO, while rGO and nZVI/rGO exhibit the shift of the peaks [41,49]. The Raman spectrum of rGO exhibited the two well-known D and G bands centered at 1349 cm^−1^ and 1593 cm^−1^. The nZVI/rGO was similar to that of rGO, with sharp D and G peaks at 1349 cm^−1^ and 1588 cm^−1^, respectively. The intensity ratio of the D to G peaks (*r* = *I*_D_/*I*_G_) is a measure of the disorder in the graphene. The *I_D_/I_G_* ratios of GO, rGO, and nZVI/rGO are 0.93, 1.135, and 1.41, respectively. The value of the *I_D_/I_G_* ratio is indicative of the size of the sp^2^ domains, which is attributed to the changes in the GO structures with an increase in the values’ ratio.

**Figure 6:**
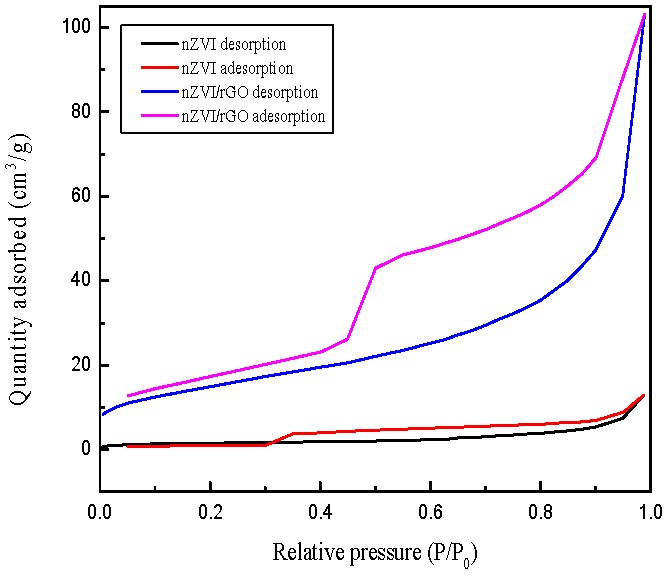
The adsorption/desorption isotherms of nZVI and nZVI/rGO.

Figure 6 shows the adsorption-desorption isotherms of the nZVI and nZVI/rGO. The BET specific surface areas of the nZVI and nZVI/rGO were 5.31 and 54.16 m^2^/g, respectively. The specific surface area of the nZVI/rGO was significantly higher than that of the nZVI, which plays a significant role in the adsorption of contaminants.

**Figure 7:**
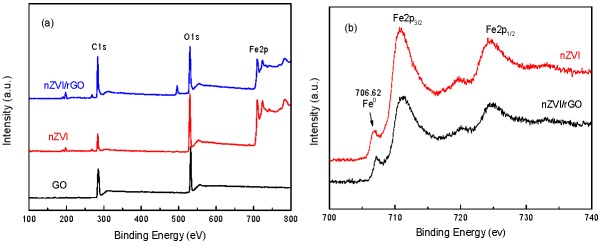
X-ray photoelectron spectra (XPS) analyses of nZVI and nZVI/rGO: wide scan (**a**); High resolution spectra of Fe2p in lab made nZVI (**b**).

In the X-ray photoelectron spectra (XPS), the peaks located at 284.6 eV, 531.8 eV, and 711.3 eV (Figure 7a) are assigned to the characteristic peaks of C1s, O1s, and Fe2p, respectively. In the spectrum of Fe2p (Figure 7b), a feature peak of Fe^0^ can be found at around 706.62 eV, suggesting that Fe^0^ exists on the rGO surface. The peaks of Fe2p_3/2_ and Fe2p_1/2_ are approximately at 710.68 eV and 724.57 eV for γ-Fe_2_O_3_, which indicated that the surface oxidization of nZVI particles is inevitable during the synthesis processes [50,51,52,53]. The peak of Fe^0^ is very small relative to the main Fe2p3/2 peak at higher energy, which also suggests that the surface of nZVI is heavily oxidized.

### 3.2. RSM-BBD Modeling

In order to investigate the combined effect of the factors (initial pH, initial concentration, temperature, and contact time), we performed statistically designed experiments at three levels. As shown in Table 2, the design variable matrix of the experimental plan consisted of 29 runs, and the experiments’ results for the removal efficiency of Rh B onto nZVI/rGO have good agreement with the predicted values. In addition, the center point (0, 0, 0, 0) was repeated five times and similar results were obtained, indicating a satisfactory reproducibility of the data.

**Figure 8:**
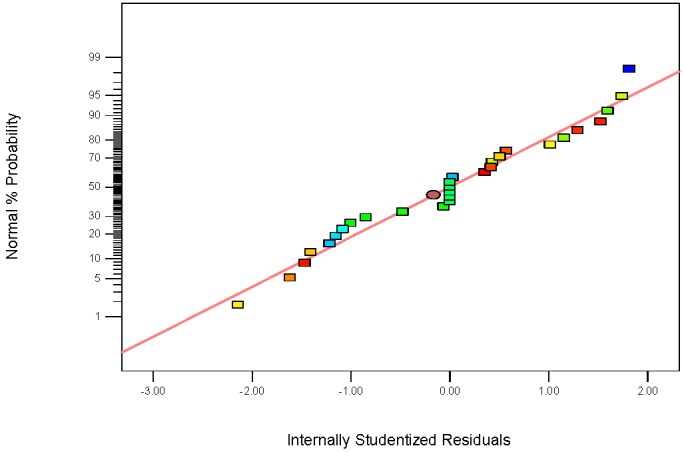
Normal plot of studentized residuals versus normal percent probability.

**Figure 9:**
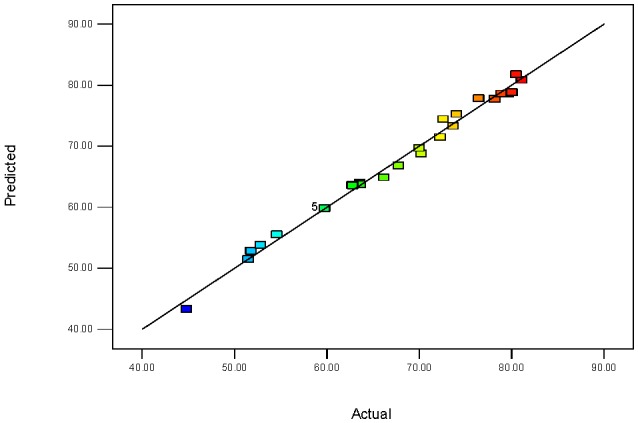
The relationship between the predicted and actual values for the Box-Behnken design (BBD).

The normal probability plot of the residuals has been provided in Figure 8. According to this graph, it is evident that the data from the experiments come from a normally distributed population due to the fact that the points fall approximately close to the straight line, which showed the homogeneously distributed data on either side of the line, indicating the suitability of the model. In Figure 9, the actual and predicted values were close for the removal efficiency of Rh B. It is illustrated that the experimental results are in good agreement with the predicted values, which is able to give a good prediction of the response for the removal process.

The results of ANOVA are summarized in Table 3. Statistical significance of the model, individual factors, their squares, and interactions were estimated from their *F*-values and *p*-values [35]. The ANOVA for the response surface quadratic model gives an *F*-value of 119.07, which implies that the terms have a significant effect on the response in the model. As can be observed in Table 3, the *p*-values are all less than 0.050 for initial pH (*A*), initial concentration (*B*), and temperature (*C*), indicating that they are statistically significant. Meanwhile, the interaction effects of initial pH and initial concentration (*AB*), initial pH (*A*^2^), initial concentration (*B*^2^), and temperature (*C*^2^) and contact time (D^2^) are also at the 95% confidence level. Among the individual process variables, the most important was the initial concentration (*B*), followed by initial pH and temperature. The model also gives the *R*^2^ value of 0.9919 and the adjusted-*R*^2^ value of 0.9838, which show that the model is accurate and reliable.

The response function with determined coefficients for the removal of Rh B is given below:

*Y* = 59.79 − 6.16*A* − 12.59*B* − 1.38*C* − 0.95*D* − 4.61*AB* + 1.30*AC* + 0.75*AD* + 1.87*BC* − 1.07*BD* − 0.04*CD* + 5.38*A*^2^ + 1.51*B*^2^ + 4.60*C*^2^ + 4.78*D*^2^ (5)

**Figure 10:**
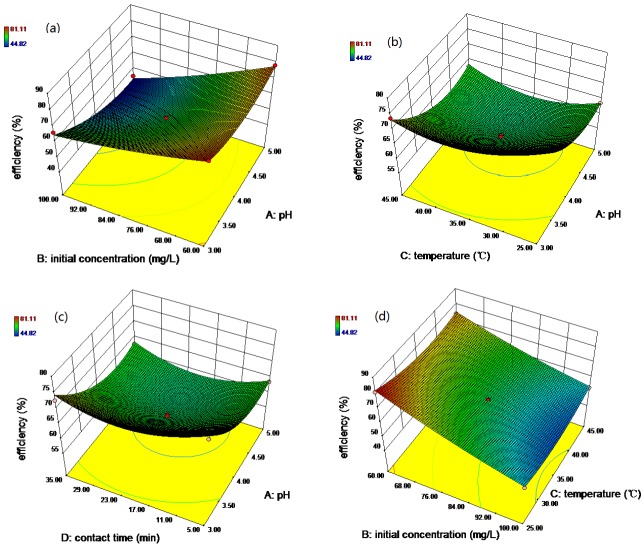

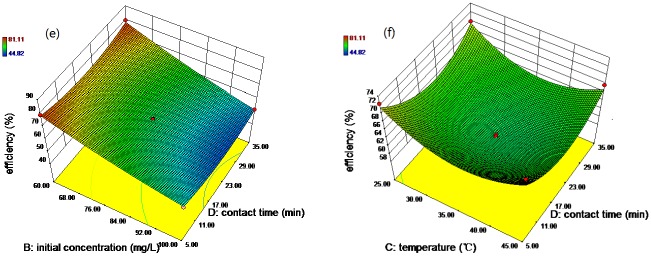
The 3D plots showing the effect of initial pH and initial concentration (**a**); initial pH and temperature (**b**); initial pH and contact time (**c**); initial concentration and temperature (**d**); initial concentration and contact time (**e**); and temperature and contact time (**f**) on the removal of Rh B.

To better study and understand the effects of the independent variables and their interactions on the response, 3D response surface plots are employed and the results are shown in Figure 10. From the ANOVA analysis, the initial pH and initial concentration are considered to be most effective in the adsorption processes. Figure 10a shows the interactive effect between the initial pH (3–5) and initial concentration (60–100 mg/L) on the removal percentage of Rh B. It is evident that the Rh B removal efficiency decreases with elevated initial concentration. Furthermore, the decrease in the adsorption of Rh B increased with the initial pH in the experimental range. The reason for this may be attributed to the surface charge of the adsorbent materials. At low initial pH, neutralization of the negative charge at the surface increases the protonation, hence exhibiting high affinity for the cationic dyes (Rh B), which are positively charged in acidic medium [2,54]. The interactive effects between the initial pH and temperature, initial pH and contact time, initial concentration and temperature, initial concentration and contact time, and temperature and contact time are shown in Figure 10.

### 3.3. ANN Modeling

**Figure 11:**
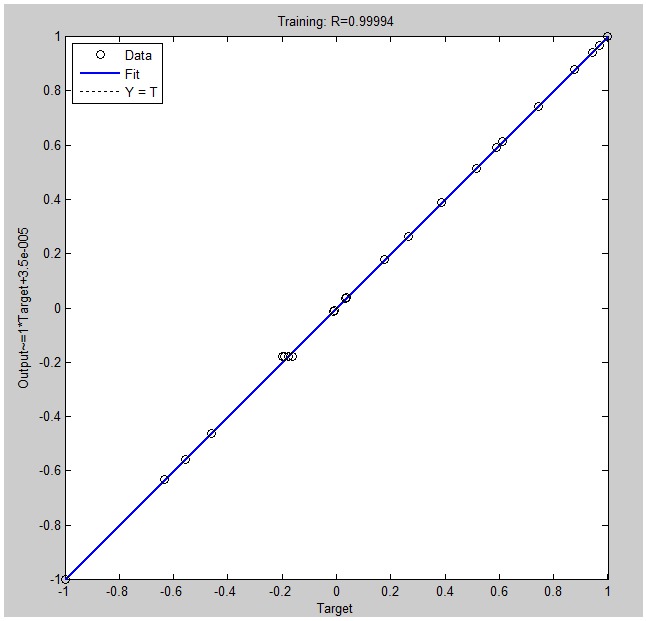
Regression plot (Actual vs. Predicted) using the ANN model.

**Figure 12:**
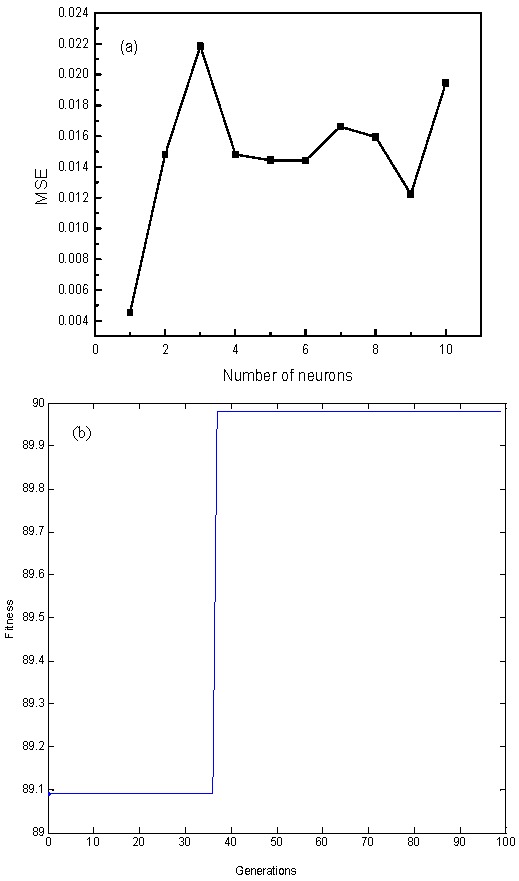
Relationship between the number of neurons and mean square error (MSE) (**a**); Evolvement of fitness with 100 generations (**b**).

The data obtained from a total of 29 experiments based on the RSM design were used for the establishment of an artificial neural network model. The ANN model was developed using the back propagation (BP) algorithm, and the prediction model had four inputs (initial pH, initial concentration, temperature, and contact time) and one output (the removal efficiency of Rh B), respectively. The trained network gave a mean square error (MSE) of 0.0045 with a regression coefficient of 0.9994. The regression plots of the trained network are shown in Figure 11. In addition, MSE was used as a criterion in relation to the number of neurons in the hidden layer, which had a direct effect on the fitting performance of the network. As shown in Figure 12a, one neuron in the hidden layer was found to be the most suitable structure to best represent the Rh B removal, because further increasing the number of neurons made the model less accurate. The predicted values of Rh B removal using the BP-ANN model are shown in Table 2. As listed in Table 2, the predicted values of the ANN model are more accurate than that of the RSM model. Moreover, the ANN model performed much better regarding the optimum removal efficiency of Rh B than the RSM model (Table 4).

### 3.4. Optimization for the Removal of Rh B by RSM and ANN-GA

Optimization of the process variables to maximize the removal of Rh B by nZVI/rGO using both the RSM and ANN-GA models are compared in Table 4. The best fitness plot achieved during the iterations of GA is shown in Figure 12b. The RSM model predicts that the removal efficiency of Rh B is 95.2% under the following conditions: initial pH of 3.00, initial concentration of 60 mg/L, temperature of 25 °C, and contact time of 5.30 min. According to the ANN-GA model, the efficiency of Rh B removal is 90.0% and corresponds to an initial pH of 3.20, initial concentration of 60 mg/L, temperature of 27 °C, and contact time of 6.00 min. Furthermore, it can be obtained that the absolute error between the experimental value and the predicted value of Rh B removal was 7.8% and 3.6%, respectively. This study suggests that ANN-GA can be considered as a more effective tool for predicting the Rh B removal, as compared with the RSM model.

### 3.5. Equilibrium Adsorption Isotherm and Kinetics Studies

**Figure 13:**
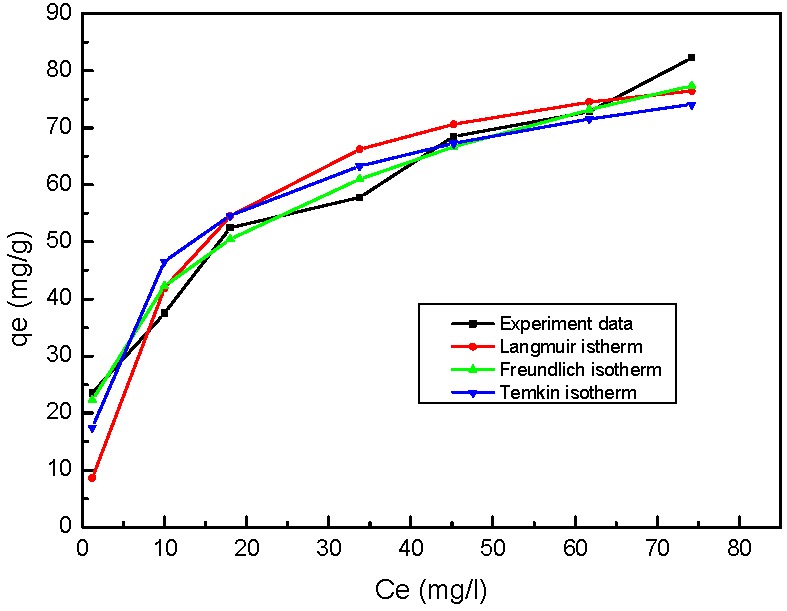
Adsorption isotherm of Rh B by nZVI/rGO. (initial pH = 5.0; nZVI/rGO dosage = 40 mg; temperature = 25 °C; and Rh B concentration = 60 mg/L).

In this study, the Langmuir, Freundlich, and Temkin isotherm models were used to fit the experimental data of adsorption equilibrium of Rh B onto the nZVI/rGO. The Langmuir isotherm is valid for monolayer adsorption onto the outer surface of the adsorbent [44]. Different from the Langmuir isotherm, the Freundlich isotherm is commonly used to describe the adsorption characteristics for a heterogeneous surface [3,55]. The Temkin isotherm contains a factor that explicitly takes into the account the adsorbent-adsorbate interactions [55,56]. The isotherm plots for Rh B adsorption are shown in Figure 13, and the results are presented in Table 5. As seen from Table 5, the regression coefficients (*R*^2^) of the Langmuir, Freundlich, and Temkin models were 0.9670, 0.9773, and 0.9122, respectively. This indicated that the Freundlich model was the most suitable for describing the process of sorption by nZVI/rGO in the present study. For the Freundlich model, the constant *K*f is an approximate indicator of adsorption capacity. The value of the parameter *K*_f_ is 21.11, which is higher than that reported by a previous study [57]. Furthermore, 1/*n* is the heterogeneity factor. The slope (1/*n*) of less than one indicates chemisorption, and more than one indicates co-operative adsorption [58]. Thereby the adsorption of Rh B on nZVI/rGO is by chemisorption (Table 5). Based on the Langmuir isotherm, the maximum monolayer sorption capacity (*q*_m_) value of Rh B was 87.72 mg/g, which was close to the experimental value (85.00 mg/g). The adsorption capacity of the nZVI/rGO composites for Rh B was higher than those of other adsorbents, such as perlite [57], sodium montmorillonite [59], and hexadecyltrimethylammonium bromide-modified *V. volvacea* (HMV) [60]. In addition, the value of R_L_ was in the range of 0.0641–0.3539, which confirmed that the adsorption is favorable for Rh B.

**Figure 14:**
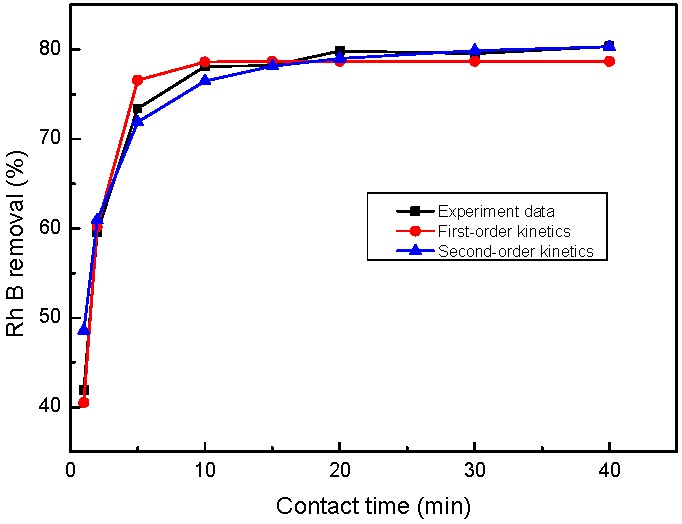
Time dependent study of Rh B removal by nZVI/rGO. (initial pH = 5.0; nZVI/rGO dosage = 40 mg; temperature = 25 °C; Rh B concentration = 60 mg/L).

The adsorption kinetics can be described by the pseudo-first order kinetic model and the pseudo-second order kinetic model. The two kinetic models were fit to the experimental data for estimating the possible mechanism and rate of Rh B adsorption onto nZVI/rGO. The values of the parameters and R^2^ are shown in Table 6. It can be seen that the pseudo-second order model can describe the Rh B removal processes significantly better than the pseudo-first order kinetic model because of its higher R^2^ value. Figure 14 shows that equilibrium is achieved for the full removal process within 20 min for the nZVI/rGO composites, with the removal capacity of 57.50 mg/g. Moreover, its calculated adsorption capacity reasonably fit the experimental data, which provided further evidence for the use of the pseudo second order kinetic model.

where *q*_e_ and *q*_t_ are the amounts of Rh B removal at the equilibrium time and time *t* (mg/g); *k*_1_ (1/min) and *k*_2_ (g/mg·min) represent the equilibrium rates for the pseudo first order kinetic model and pseudo second order kinetic model, respectively.

## 4. Conclusions

The nano-composite absorbent nZVI/rGO was employed in this study to investigate the removal capacities for Rh B in aqueous solutions. The synthesized materials of nZVI and nZVI/rGO were characterized by SEM, XRD, Raman spectroscopy, N_2_-sorption, and XPS. The RSM and ANN-GA models were developed to determine the optimum process conditions for the removal efficiency of Rh B. Both models provide a good quality prediction for the adsorption behavior. Obviously, based on the experimental data with the optimum conditions, the ANN-GA model was found to be more accurate in predicting the removal efficiency of Rh B than the RSM model (absolute error of 3.6% and 7.8% for ANN-GA and RSM, respectively). It is noted that the most important individual process variable was the initial concentration via ANOVA analysis for the RSM model. The Freundlich model and the pseudo-second order kinetic model were more suited for describing the process of Rh B removal by nZVI/rGO. This work provides new insights for developing novel adsorbents to remove Rh B, while the thermodynamics of Rh B removal still require further study. In addition, it would also be useful to evaluate the nZVI/rGO sorption efficiency of multiple dyes from aqueous solutions and whether Rh B affects this sorption. This will pave the way for practical applications in the treatment of noxious dye containing wastewater.

## Figures and Tables

**Table 1:** Levels of variables chosen for the Box-Behnken design.

Variable	Unit	Factors	Level		
			Low (−1)	Middle (0)	High (+1)
Initial pH		*A*	3	4	5
Initial concentration	mg/L	*B*	60	80	100
Temperature	°C	*C*	25	35	45
Contact time	min	*D*	5	20	35

**Table 2:** Experimental and predicted values of Rhodamine B (Rh B) removal (%).

Run	*A*	*B* (mg/L)	*C* (°C)	*D* (min)	Actual (%)	Predicted (%)	
						RSM	ANN
1	3	80	35	5	76.5	77.8	76.5
2	4	100	35	5	54.6	55.5	54.6
3	4	80	35	20	59.5	59.5	59.7
4	5	100	35	20	44.8	43.3	44.8
5	5	80	25	20	63.6	63.7	63.6
6	4	60	25	20	80.5	81.7	80.5
7	5	80	35	5	63.6	64.0	63.6
8	3	100	35	20	66.2	64.9	66.2
9	3	80	45	20	73.7	73.3	73.7
10	4	80	35	20	60.0	59.5	59.7
11	4	80	35	20	59.8	59.5	59.7
12	5	80	35	35	62.8	63.6	62.8
13	4	60	45	20	74.1	75.2	74.1
14	5	80	45	20	62.8	63.5	62.8
15	4	80	35	20	59.8	59.8	59.7
16	4	80	25	5	72.3	71.4	72.3
17	4	80	25	35	70.0	69.6	70.0
18	4	60	35	5	78.9	78.5	78.9
19	4	60	35	35	80.1	78.8	80.0
20	4	100	35	35	51.5	51.5	51.5
21	3	60	35	20	81.1	80.8	81.1
22	4	80	45	35	67.77	66.81	67.8
23	4	80	35	20	59.37	59.51	59.7
24	4	100	45	20	52.84	53.8	52.8
25	5	60	35	20	78.2	77.72	78.6
26	4	100	25	20	51.8	52.81	54.6
27	3	80	25	20	79.68	78.6	76.1
28	3	80	35	35	72.62	74.41	69.1
29	4	80	45	5	70.23	68.78	72.3

Note: RSM: response surface methodology.

**Table 3:** Pareto analysis of variance (ANOVA) of the second-order polynomial equation.

Source	Sum of Squares	df	Mean Square	*F*-Value	*p*-Value
Model	2870.1	14	205.01	119.07	<0.0001
*A*	455.35	1	455.35	264.48	<0.0001
*B*	1903.1	1	1903.1	1105.38	<0.0001
*C*	22.77	1	22.77	13.23	0.0027
*D*	10.74	1	10.74	6.24	0.0256
*AB*	85.19	1	85.19	49.48	<0.0001
*AC*	6.71	1	6.71	3.90	0.0685
*AD*	2.28	1	2.28	1.32	0.2691
*BC*	14.03	1	14.03	8.15	0.0127
*BD*	4.60	1	4.60	2.67	0.1244
*CD*	6.40×10^−3^	1	6.40×10^−3^	3.72×10^−3^	0.9522
*A*2	197.47	1	197.47	114.69	<0.0001
*B*2	17.61	1	17.61	10.23	0.0064
*C*2	145.81	1	145.81	84.69	<0.0001
*D*2	156.93	1	156.93	91.15	<0.0001
Residual	24.1	14	1.72		
Lack of Fit	23.33	10	2.33		
Pure Error	0.77	4	0.19		
Cor Total	2894.2	28			

**Table 4:** Comparison of the optimal process conditions and optimum removal efficiency for Rh B by response surface methodology (RSM) and artificial neural network hybridized with genetic algorithm (ANN-GA) optimization.

Process Variable	RSM Optimization	ANN-GA Optimization
Initial pH	3.00	3.20
Initial concentration (mg/L)	60.00	60.00
Temperature (°C)	25.00	27.00
Contact time (min)	5.30	6.00
Efficiency, model (%)	95.2	90.0
Efficiency, actual (%)	87.4	86.4

**Table 5:** Langmuir, Freudlich, and Temkin isotherm parameters for the removal of Rh B by nZVI/rGO.

Isotherms	Equation	Parameters	Value of Parameters
Langmuir	$\frac{C_{e}}{q_{e}}$ = $\frac{1}{qmK_{L}}$ + $\frac{C_{e}}{q_{m}}$ *R_L_* = 1/(1 + *K_L_C*_0_)	*K_L_* (L/mg)	0.0913
		*q_m_* (mg/g)	87.72
		*R*^2^	0.9670
		*R_L_*	0.0641–0.3539
Freundlich	$\log q_{e}=\log K_{f}+\frac{1}{n}\log C_{e}$	*K_f_* (mg/g)	21.11
		1/*n*	0.3016
		*R*^2^	0.9773
Temkin	*q_e_* = *B*ln*A* + *B*ln*C_e_*	*A* (L/g)	2.988
		*B*	13.711
		*R*^2^	0.9122

**Table 6:** Kinetic parameters for the removal of Rh B on nZVI/rGO.

Model	Equation	Parameters	Value of Parameters
First-order kinetics	$\log(q_{e}-q_{t})=\log q_{e}-k_{1}t/2.303$	*k*_1_ (1/min)	0.72
		*q_e_* (mg/g)	56.65
		*R*^2^	0.9866
Second-order kinetics	$t/q_{t}=1/k_{2}{q^{2}}_{e}+t/q_{e}$	*k*_2_ (g/mg·min)	2.49 × 10^−2^
		*q_e_* (mg/g)	58.82
		*R*^2^	0.9999

## Supplementary Materials

Supplementary File 1

## References

[1] Hou M.F., Ma C.X., Zhang W.D., Tang X.Y., Fan Y.N., Wan H.F. (2011). Removal of rhodamine B using iron-pillared bentonite. J. Hazard. Mater..

[2] Singh K.P., Gupta S., Singh A.K., Sinha S. (2010). Experimental design and response surface modeling for optimization of Rhodamine B removal from water by magnetic nanocomposite. Chem. Eng. J..

[3] Lei D.Y., Li B., Wang Q., Wu B., Ma L., Xu H. (2015). Removal of Neutral Red from aqueous solution using Pleurotus ostreatus nanoparticles by response surface methodology. Desalin. Water Treat..

[4] Dong X., Ding W., Zhang X., Liang X. (2007). Mechanism and kinetics model of degradation of synthetic dyes by UV–vis/H_2_O_2_/ferrioxalate complexes. Dyes Pigm..

[5] Wong Y., Yu J. (1999). Laccase-catalyzed decolorization of synthetic dyes. Water Res..

[6] Banate I.M., Nigam P., Singh D., Marchant R. (1996). Microbial decolorization of textile dye containing effluents: A review. Bioresour. Technol..

[7] Slimane M., Oualid H., Fethi S., Mahdi C., Christian P. (2010). Influence of bicarbonate and carbonate ions on sonochemical degradation of rhodamine B in aqueous phase. J. Hazard. Mater..

[8] Lu L., Zhao M., Liang S.C., Zhao L.Y., Li D.B., Zhang B.B. (2009). Production and synthetic dyes decolourization capacity of a recombinant laccase from Pichia pastoris. J. Appl. Microbiol..

[9] Salleh M.A.M., Mahmoud D.K., Karim W.A.W.A., Idris A. (2011). Cationic and anionic dye adsorption by agricultural solid wastes: A comprehensive review. Desalination.

[10] Inbaraj B.S., Chien J.T., Ho G.H., Yang J., Chen B.H. (2006). Equilibrium and kinetic studies on sorption of basic dyes by a natural biopolymer poly(gamma-glutamic acid). Biochem. Eng. J..

[11] Kadirvelu K., Karthika C., Vennilamani N., Pattabhi S. (2005). Activated carbon from industrial solid waste as an adsorbent for the removal of Rhodamine-B from aqueous solution: Kinetic and equilibrium studies. Chemosphere.

[12] Nestmann E.R., Douglas G.R., Matula T.I., Grant C.E., Kowbel D.J. (1979). Mutagenic activity of rhodamine dyes and their impurities as detected by mutation induction in Salmonella and DNA damage in Chinese hamster ovary cells. Cancer Res..

[13] Bulut E., Özacar M., Şengil İ.A. (2008). Equilibrium and kinetic data and process design for adsorption of Congo Red onto bentonite. J. Hazard. Mater..

[14] Saif S., Tahir A., Chen Y. (2016). Green synthesis of iron nanoparticles and their environmental applications and implications. Nanomaterials.

[15] Li L.Y., Hu J.W., Shi X.D., Fan M.Y., Luo J., Wei X.H. (2016). Nanoscale zero-valent metals: A review of synthesis, characterization and applications to environmental remediation. Environ. Sci. Pollut. Res..

[16] Fan M.Y., Li T., Hu J.W., Cao R.S., Wu Q., Wei X.H., Li L.Y., Shi X.X., Ruan W.Q. (2016). Synthesis and characterization of reduced graphene oxide-supported nanoscale zero-valent iron (nZVI/rGO) composites used for Pb(II) removal. Materials.

[17] Janiak C. (2015). Metal nanoparticle synthesis in ionic liquids. Top. Organoment. Chem..

[18] Khan M.S., Ahmad A., Bangash F.U.K., Shah S.S., Khan P. (2013). Removal of Basic Dye from Aqueous Solutions Using Nano Scale Zero Valent Iron (NZVI) as Adsorbent. J. Chem. Soc. Pak..

[19] Joo S.H., Feitz A.J., Waite T.D. (2004). Oxidative degradation of the carbothioate herbicide molinate, using nanoscale zero-valent iron. Environ. Sci. Technol..

[20] Geng B., Jin Z.H., Li T.L., Qi X.H. (2009). Preperation of chitosan-stabilized Fe^0^ nanopartiicles for removel of hexavalent chromium in water. Sci. Total Environ..

[21] Zhu H.J., Jia Y.F., Wu X., Wang H. (2009). Removal of arsenic from water by supported nano zero-valent iron on activated carbon. J. Hazard. Mater..

[22] Zhang X., Lin S., Chen Z.L., Megharaj M., Naidu R. (2011). Kaolinite-supported nanoscale zero-valent iron for removal of Pb^2+^ from aqueous solution: Reactivity, characterization and mechanism. Water Res..

[23] Li J., Chen C.L., Zhu K.R., Wang X.K. (2016). Nanoscale zero-valent iron particles modified on reduced graphene oxides using a plasma technique for Cd(II) removal. J. Taiwan Inst. Chem. Eng..

[24] Crane R.A., Scott T. (2014). The removal of uranium onto carbon-supported nanoscale zero-valent iron particles. J. Nanopart. Res..

[25] Wang C., Luo H.J., Zhang Z.L., Wu Y., Zhang J., Chen S.W. (2014). Removal of As(III) and As(V) from aqueous solutions using nanoscale zero valent iron-reduced graphite oxide modified composites. J. Hazard. Mater..

[26] Ahmad A., Gu X.G., Li L., Lv S.G., Xu Y.S., Guo X.H. (2015). Efficient degradation of trichloroethylene in water using persulfate activated by reduced graphene oxide-iron nanocomposite. Environ. Sci. Pollut. Res..

[27] Du J.J., Zhou Q.X. (2014). Preliminary study on effects of nanoscale amendments on hyperaccumulator Indian Marigold grow on co-contaminated soils. Adv. Mater. Res..

[28] Duarte B.P.M., Saraiva P.M. (2003). Hybrid models combining mechanistic models with adaptive regression splines and local stepwise regression. Ind. Eng. Chem. Res..

[29] Achenie L., Butkus M.A., Grasso D., Schulthess C.P., Morris T., Hyde J. (2001). A comparative study of neural network and mechanistic models for surface complexation. Adv. Environ. Res..

[30] Sahu J.N., Acharya J., Meikap B.C. (2009). Response surface modeling and optimization of chromium(VI) removal from aqueous solution using Tamarind wood activated carbon in batch process. J. Hazard. Mater..

[31] Kaan Y., Sevgi D. (2008). Artificial neural network (ANN) approach for modeling of Pb(II) adsorption from aqueous solution by Antep pistachio (*Pistacia Vera* L.) shells. J. Hazard. Mater..

[32] Alam Z., Muyibi S.A., Toramae J. (2007). Statistical optimization of adsorption processes for removal of 2,4-dichlorophenol by activated carbon derived from oil palm empty fruit bunches. J. Environ. Sci..

[33] Arsand D.R., Kümmerer K., Martins A.F. (2013). Removal of dexamethasone from aqueous solution and hospital wastewater by electrocoagulation. Sci. Total Environ..

[34] Son J., Vavra J., Forbes V.E. (2015). Effects of water quality parameters on agglomeration and dissolution of copper oxide nanoparticles (CuO-NPs) using a central composite circumscribed design. Sci. Total Environ..

[35] Avramović J.M., Veličković A.V., Stamenković O.S., Rajković K.M., Milić P.S., Veljković V.B. (2015). Optimization of sunflower oil ethanolysis catalyzed by calcium oxide: RSM versus ANN-GA. Energy Convers. Manag..

[36] Rajendra M., Jena PC., Raheman H. (2009). Prediction of optimized pretreatment process parameters for biodiesel production using ANN and GA. Fuel.

[37] Çelekli A., Bozkurt H., Geyik F. (2012). Use of artificial neural networks and genetic algorithms for prediction of sorption of an azo-metal complex dye onto lentil straw. Bioresour. Technol..

[38] Huang M., Ma Y., Wan J., Zhang H., Wang Y., Chen Y., Yoo C., Guo W. (2011). A hybrid genetic-neural algorithm for modeling the biodegradation process of DnBP in AAO system. Bioresour. Technol..

[39] Hummers W.S., Offeman R.E. (1958). Preparation of graphitic oxide. J. Am. Chem. Soc..

[40] Marcano D.C., Kosynkin D.V., Berlin J.M., Sinitskii A., Sun Z., Slesarev A., Alemany L.B., Lu W., Tour J.M. (2010). Improved synthesis of graphene oxide. ACS Nano.

[41] Jabeen H., Kemp K.C., Chandra V. (2013). Synthesis of nano zerovalent iron nanoparticles-Graphene composite for the treatment of lead contaminated water. J. Environ. Manag..

[42] Li B.J., Cao H.Q., Yin G., Lu Y.X., Yin J.F. (2011). Cu_2_O@reduced Graphene oxide composite for removal of contaminants from water and supercapacitor. J. Mater. Chem..

[43] Ashrafi S.D., Kamani H., Jaafari J., Mahvi A.H. (2016). Experimental design and response surface modeling for optimization of fluoroquinolone removal from aqueous solution by NaOH-modified rice husk. Desalin. Water Treat..

[44] Madala S., Mudumala V.N.R., Vudagandla S., Abburi K. (2015). Modified leaf biomass for Pb(II) removal from aqueous solution: Application of response surface methodology. Ecol. Eng..

[45] Tripathi P., Srivastava V.C., Kumar A. (2009). Optimization of an azo dye batch adsorption parameters using Box-Behnken design. Desalination.

[46] Zhang Y., Pan B. (2014). Modeling batch and column phosphate removal by hydrated ferric oxide-based nanocomposite using response surface methodology and artificial neural network. Chem. Eng. J..

[47] Winiczenko R., Górnicki K., Kaleta A., Janaszek-Mańkowska M. (2016). Optimisation of ANN topology for predicting the rehydrated apple cubes colour change using RSM and GA. Neural Comput. Appl..

[48] Pal M.P., Vaidya B.K., Desai K.M., Joshi R.M., Nene S.N., Kulkarni B.D. (2009). Media optimization for biosurfactant production by *Rhodococcus erythropolis* MTCC 2794: Artificial intelligence versus a statistical approach. J. Ind. Microbiol. Biotechnol..

[49] Tuinstra F., Koenig J.L. (1970). Raman spectrunm of graphite. J. Chem. Phys..

[50] Biesinger M.C., Payne B.P., Grosvenor A.P., Lau L.W.M., Gerson A.R., Smart R.St.C. (2011). Resolving surface chemical states in XPS analysis of first row transition metals, oxides and hydroxides: Cr, Mn, Fe, Co and Ni. Appl. Surf. Sci..

[51] He D., Ma J., Collins R.N., Waite T.D. (2016). Effect of structural transformation of nanoparticulate zero-valent iron on generation of reactive oxygen species. Environ. Sci. Technol..

[52] Chandra V., Park J., Chun Y., Lee J.W., Hwang I.C., Kim K.S. (2010). Water-dispersible magnetite-reduced graphene oxide composites for arsenic removal. ACS Nano.

[53] Manning B.A., Kiser J.R., Kwon H., Kanel S.R. (2007). Spectroscopic investigation of Cr(III)- and Cr(VI)-treated nanoscale zerovalent iron. Environ. Sci. Technol..

[54] Jain R., Mathur M., Sikarwar S., Mittal A. (2007). Removal of the hazardous dye rhodamine B through photocatalytic and adsorption treatments. J. Environ. Manag..

[55] Dada A.O., Olalekan A.P., Olatunya A.M., Dada O. (2012). Langmuir, Freundlich, Temkin and Dubinin-Radushkevich isotherms studies of equilibrium sorption of Zn^2+^ unto phosphoric acid modified rice husk. J. Appl. Chem..

[56] Mohammadi M., Hassani A.J., Mohamed A.R., Najafpour G.D. (2010). Removal of Rhodamine B from aqueous solution using palm shell-based activated carbon: Adsorption and kinetic studies. J. Chem. Eng. Data.

[57] Vijayakumar G., Tamilarasan R., Dharmendirakumar M. (2012). Adsorption, Kinetic, Equilibrium and Thermodynamic studies on the removal of basic dye Rhodamine-B from aqueous solution by the use of natural adsorbent perlite. J. Mater. Environ. Sci..

[58] Patil S.P., Bethi B., Sonawane G.H., Shrivastava V.S., Sonawane S. (2016). Efficient adsorption and photocatalytic degradation of Rhodamine B dye over Bi_2_O_3_-bentonite nanocomposites: A kinetic study. J. Ind. Eng. Chem..

[59] Selvam P.P., Preethi S., Basakaralingam P., Thinakaran N., Sivasamy A., Sivanesan S. (2008). Removal of rhodamine B from aqueous solution by adsorption onto sodium montmorillonite. J. Hazard. Mater..

[60] Li Q., Tang X., Sun Y., Wang Y., Long Y., Jiang J., Xu H. (2015). Removal of Rhodamine B from wastewater by modified Volvariella volvacea: Batch and column study. RSC. Adv..

