# Addendum: Shi, X.D.; Ruan, W.Q.; Hu, J.W.; Fan, M.Y.; Cao, R.S.; Wei, X.H. Optimizing the Removal of Rhodamine B in Aqueous Solutions by Reduced Graphene Oxide-Supported Nanoscale Zerovalent Iron (nZVI/rGO) Using an Artificial Neural Network-Genetic Algorithm (ANN-GA). *Nanomaterials* 2017, *7*, 134

**DOI:** 10.3390/nano7100309

**Published:** 2017-10-08

**Authors:** Xuedan Shi, Wenqian Ruan, Jiwei Hu, Mingyi Fan, Rensheng Cao, Xionghui Wei

**Affiliations:** 1 Guizhou Provincial Key Laboratory for Information Systems of Mountainous Areas and Protection of Ecological Environment, Guizhou Normal University, Guiyang 550001, China; xuedanshi1991@163.com (X.S.); wenqianruan@yahoo.com (W.R.); fanmingyifmy@163.com (M.F.); 18230825324@163.com (R.C.); 2 Department of Applied Chemistry, College of Chemistry and Molecular Engineering, Peking University, Beijing 100871, China; xhwei@pku.edu.cn

The authors wish to make the following addendum to their paper [1]:

We need to add the following three references to Section 3.5 “Equilibrium Adsorption Isotherm and Kinetics Studies” of our recently published paper [1]: “Zur Theorie Der Sogenannten Adsorption Gelöster Stoffe”, by Lagergren, published in *Bihang till K. Svenska Vet-Akad. Handlingar*, 1898 [2]; “Adsorption of Heavy Metals from Waste Streams by Peat”, by Ho, Ph.D. Thesis, University of Birmingham, 1995 [3]; “Sorption of Dye from Aqueous Solution by Peat”, by Ho and McKay, published in *Chem. Eng. J.*, 1998 [4]. These three articles described the adsorption kinetics, i.e., the pseudo-first order kinetic model and the pseudo-second order kinetic model.

The authors would like to apologize for any inconvenience caused. The change does not affect the scientific results. The manuscript will be updated and the original will remain online on the article webpage.
